# Epilepsy in Asian countries

**DOI:** 10.1186/s42494-023-00136-1

**Published:** 2023-10-13

**Authors:** Alhamdu Adamu, Rui Chen, An Li, Guofang Xue

**Affiliations:** 1 The Second Affiliated Hospital of Shanxi Medical University, Shanxi, 030001 Taiyuan China; 2 Department of Neurology, The Second Affiliated Hospital of Shanxi Medical University, Taiyuan, 030001 China

**Keywords:** Epilepsy, Current situation, Treatment, Asia

## Abstract

Epilepsy affects 50 million people worldwide. Nearly 80% of people with epilepsy live in resource-constrained low-income and middle-income countries. In Asia, which has a population of over 4 billion or has 50% of the world's population, about 23 million people have epilepsy. In this review, we discuss the difficulties in managing epilepsy in Asia due to the limited resources. The medical expense, limited access to treatment, premature mortality, health transitions from pediatric care to adult care, and the huge population size make it challenging for epilepsy management. Even though certain countries have access to highly innovative treatments, up to 90% of patients with epilepsy do not receive proper care due to limited resources. The insufficiency of research on epilepsy in most countries makes it difficult to obtain accurate data to analyze the progress of epilepsy management. However, the current influx of research studies, acceptance of the latest international practices, and funding will contribute a long way to closing treatment gaps in communities.

## Introduction

Epilepsy is a neurological disorder that affects over 50 million people worldwide and is the most prevalent brain condition [1]. Individuals, regardless of gender, age, race, financial class, and geography, can be affected by epilepsy [2]. Epilepsy is an ancient disease with a history of over 4000 years. The inscriptions on the "Akkadian Tablet" in Mesopotamia read "his neck turns left, his hands and feet tense, his eyes wide open, and his mouth foams without consciousness [3]". Historically, the Mesopotamians believed that epilepsy was caused by evil spirits and could be treated by spiritual rituals, while the Egyptians thought that epilepsy resulted from cortical disruption.

Epilepsy involves two or more unprovoked seizures occurring at least 24 h apart [4]. Individuals with epilepsy experience abnormal neuronal activity in the brain, leading to seizures. A seizure is a paroxysmal event caused by abnormal excessive or synchronous neuronal activity in the brain [4]. The prevalence and incidence of epilepsy vary among countries and can differ between males and females, with some countries showing higher rates in males and others showing higher rates in females [5]. In addition, certain racial and ethnic groups may have higher rates of epilepsy than others. Socioeconomic factors such as poverty, malnutrition, and illiteracy are associated with a higher incidence of epilepsy in some countriess [6]. To explore the impact of socioeconomic factors on epilepsy, it is vital to distinguish between focal and generalized epilepsies.

Focal epilepsy is a spectrum of seizure disorders that affect only one hemisphere of the brain, ranging from unifocal to multifocal disorders. These disorders can present with any type of focal seizure and are diagnosed clinically, but the diagnosis is supported by an interictal electroencephalography (EEG) that detects focal epileptiform discharges [7]. Generalized epilepsy encompasses a wide range of seizure types, and a patient can exhibit any type of generalized seizures, either with motor or non-motor symptoms. The diagnosis is established based on the detection of generalized spike-wave activity on EEG combined with the patient's clinical presentation [8]. Fever, traumatic brain injury, cerebrovascular disease, drug withdrawal, and infection are some of the most common precipitating factors [2]. In one study, patients with epilepsy (PWE) experienced seizure exacerbations during the coronavirus disease 19 (COVID-19) outbreak. In these patients, stress is an independent precipitant of seizures [9]. Research has shown that patients with active epilepsy (who suffer 1-2 unprovoked seizures within 12 months and are taking medication) still experience frequent episodes [10]. Early seizures after an insult (≤ 7 days) are not considered to be epilepsy, but there is a risk of approximately 25% for developing epilepsy. Late seizures after an insult (≥ 7 days), if unprovoked, recur in 75-90% and are considered epilepsy [11]. This review seeks to examine the distinctive features of epilepsy in Asian countries, focusing on the epidemiology, risk factors, diagnostic difficulties, treatment options, and the influence of cultural beliefs and stigma on the lives of those with epilepsy. This review aims to provide a thorough understanding of the state of epilepsy in Asian countries and emphasize the need for new intervention approaches and collaborations between governments and non-governmental organizations (NGOs) to tackle the difficulties and obstacles faced by people with epilepsy in Asia.

## Epidemiology of epilepsy in Asian countries

The World Health Organization estimates that the worldwide prevalence of epilepsy is roughly 4 to 10 per 1000 [12], with disparities among different regions. On a global scale, the median prevalence of epilepsy is highest in sub-Saharan Africa (15.0/1000) and Latin America (17.8/1000), while the prevalence in Asia is comparable to that of Western countries (6 in 1000) [13]. However, the rate of occurrence varies across regions, with the lifetime prevalence of epilepsy ranging from 1.5 to 14.0 in 1000 person-years in Asia, 5.1 to 57.0 in 1000 person-years in Latin America, and 5.2 to 74.4 in 1000 person-years in sub-Saharan Africa [13]. This is likely attributed to the different population sizes between these regions and the rest of the globe.

Asia is the world's largest continent, with diverse nations in terms of culture, geography, economy, and healthcare system. Approximately 4 billion people (50% of the world’s population) live in Asia, and 23 million have epilepsy, with a prevalence of 1.5 to 14.0 per 1000 [14].

Asia has a lower incidence of epilepsy than Latin America or Africa, but a higher incidence than Western nations, likely due to endemic nervous system infections, such as neurocysticercosis, encephalitis, cerebral malaria, tuberculosis, and human immunodeficiency virus infection. Previous studies have shown that approximately 50% of patients in India and 47% in Nepal may have neurocysticercosis as the underlying cause of their epilepsy [15].

Researchers have found a high frequency of 10.7 per 1000 from a location with a high incidence of neurocysticercosis in Vietnam. Apart from endemic nervous system infections, other causes include stroke, neonatal injuries, and traumatic brain injury. However, the complete causes of epilepsy remain largely unknown [16]. In Asia, the International League Against Epilepsy (ILAE) classification reports that generalized seizures can be found in the range of 50-69%, while focal seizures are present in 31-50%. Symptomatic epilepsy is seen at a rate of 22-53%, idiopathic epilepsy at 4-42%, and unknown epilepsy at 13-60% [8].

Environmental risk factors and healthy lifestyle management can affect most demographic characteristics. Many community factors have hindered the diagnosis and treatment of epilepsy. Investigations have shown that misconceptions about epilepsy, economic hardship, stigma, as well as unavailability of health workers and drugs have been significant factors affecting early diagnosis and treatment in Asia [17]. In Korea, the incidence and prevalence of epilepsy have increased due to various factors, with the primary reason being the aging of the population [18]. A 14-year follow-up Cardiovascular Health Study revealed an increase in the prevalence of epilepsy from 3.7% to 5.4%, and a history of stroke was associated with a higher risk of developing epilepsy. The increases in both the incidence and prevalence of epilepsy may have been attributed to the increases in the numbers of older adults and those suffering from chronic diseases of the central nervous system [19] Table 1.

**Table 1:** The prevalence and incidence of epilepsy in some Asian countries [13, 20, 21]

Countries	Prevalence	Incidence
China	4.6-7/1000	28.8-30/100000
Japan	2.7-4/1000	24-53/100000
India	3-11.9/1000	38-60/100000
South Korea	4.8/1000	35.4/100000
Indonesia	5.6/1000	50/100000
Saudi Arabia	6.54/1000	*Data not available*
Turkey	7-12/1000	*Data not available* 37.59/100000^a^
Thailand	7.2/1000	7.27/100000
Bangladesh	8.4/1000	*Data not available*
Iran	16.6/1000	*Data not available*
Kazakhstan	3.14-4.95/1000	*Data not available*
Qatar	6.54/1000	50-61/100000
Myanmar	1.1/1000	*Data not available*
Nepal	7.3/1000	*Data not available*
Cambodia	5.8/1000	*Data not available*
Laos	7.7/1000	*Data not available*
Palestine	*Data not available*	11.4/100000

^a^Indicates regional incidence (Central Anatolia, Turkey)

## Cormobidities associated with epilepsy in Asian countries

Comorbidity refers to two or more medical conditions that occur in an individual [22]. A lesser-acknowledged aspect of epilepsy in Asia is the high occurrence of accompanying conditions, particularly depression and anxiety. It is estimated that up to 60% of people with epilepsy also suffer from depression and anxiety [23]. These comorbidities have a major effect on the quality of life (QoL) of epilepsy patients, therefore should be addressed and managed. Misperceptions of epilepsy have hindered epilepsy diagnosis. In Asia, common misconceptions about epilepsy include that seizures are hereditary, contagious, and can be transmitted through saliva; anyone can become epileptic when in contact with someone having epileptic seizures [24]. According to a study conducted in South Korea, some people believe that exercise could cause seizures. However, investigations have shown that activities seldom cause seizures and instead provide moderate protection from seizure while reducing anxiety and despair [25]. However, as epilepsy patients are prone to accidents, precautions should be taken.

In addition, some people believe that some antiseizure medications (ASMs) could impair memory as they may cause sleepiness, concluding that ASMs could damage the brain.

According to previous studies, patients with idiopathic epilepsy may have decreased empathic ability [26]. A case–control study conducted in Turkey revealed that 28.6% of the participants with epilepsy had anxiety symptoms, and seizure frequency was an independent predictor [27].The suicide rate among epilepsy patients is three times higher than that among the general population, and mental illness is the strongest predictor of suicide [28]. Stigma has adverse psychological and societal effects on epilepsy patients. Stigma comprises three stages shown below.

1. *Internalized:* people with mental illness internalize public negative perceptions and believe them to be true about themselves. This process is referred to as cognitive or emotional absorption [29].
2. *Interpersonal:* The patients encounter stigmatization from close relatives [30].
3. *Institutional*: The patients encounter covert stigmatization in the workplace and schools. As a result, some individuals keep their health concerns a secret from coworkers to maintain their careers [31].

In this three-stage system, patient information of having epilepsy is effectively disseminated from the patient's close family members within a community to institutions. This illness was thought to be caused by supernatural creatures in the past [32, 33]. Most rural populations, particularly in low- and middle-income nations, continue to have this misconception and consider epilepsy to be contagious. Due to these factors, PWE in families are emotionally draining [34]. There is a high prevalence of depression among PWE. A systematic review and meta-analysis reported a prevalence of 23.1% (95% confidence interval [CI]: 20.6–28.31%) for active depression in PWE, which is much higher than that in the general population. Children with epilepsy are five times more prone to suicide than those without epilepsy [35]. A study conducted in the United Arab Emirates (UAE) showed very limited general knowledge of and a mostly negative attitude toward epilepsy. Additionally, a research project found that people with epilepsy have higher levels of depression and anxiety than those without the condition [36]. A study conducted in western China showed that children with epilepsy commonly experience negative emotions like depression. In children, depression can manifest as mood fluctuations, impulsiveness, low self-esteem, self-harm and suicide attempts, and abdominal discomfort, which can have detrimental effects on both patients and their families [37]. A research in Bahrain found that the education level predicts death, with higher education level linked to less anxiety. Patients with lower education levels are more anxious, likely due to their less knowledge about the illness and poorer seizure management [38]. An epidemiological study in South Kazakhstan showed that only 22.66% of PWE in the urban district and 11.88% of PWE in the rural district were employed or studying [39]. PWEs who are married, enrolled in college, and find employment in Asia (marriages are forbidden and annulled because of these conditions) are likely to have better disability status [40]. Even in Malaysia with a robust economy, a survey of 250 people with epilepsy revealed that 20% were unemployed and 10.4% were employed part-time. In comparison to their siblings, they were more likely to be unemployed, unmarried, have a low level of education, and have a low income, resulting in a low level of social security [13].

Stigma remains a significant issue, even in contemporary Asian cities. In a survey conducted in Hong Kong, China, 94.1% of participants agreed that people with epilepsy could marry. However, only 67.7% would permit their child to marry a person with epilepsy, while 72% thought that pregnancy is acceptable [13]. In a questionnaire survey conducted in Jordan, the participants had a less positive attitude towards marriage to someone with epilepsy. This may be explained by the traditional customs and strong cultural values and beliefs concerning marriage Jordanians have may be the source of this unfavorable attitude [41]. A study conducted in the UAE uncovered that the general knowledge of epilepsy was very limited, and this was accompanied by a mostly negative attitude. Additionally, a study found that people with epilepsy had higher levels of depression and anxiety than those without [36].

The names of epilepsy in many Asian languages are one variable that affects epileptic stigma. In Japan, it is called "Tankan", meaning madness, while in Korea, it is called "Gan-zil," meaning "mad sickness". Chinese terms for epilepsy include " diān xián", "madness" and " yáng diān fēng", "goat madness". Due to the influence of Traditional Chinese Medicine, the names of epilepsy in some East and Southeast Asian languages also conjure up images of madness and animals [42]. The Malay people, primarily Muslims, also consider epilepsy spiritually impure (Gila babi, mad pig illness), which adds to the stigma attached to epileptic sufferers [42].

To reduce the stigma associated with epilepsy, the names given to epilepsy have been changed to neutral terminology in Malay in Malaysia, Mandarin in China, and Korean in South Korea.

Epilepsy is becoming more widely recognized and accepted, but dissemination knowledge about this condition is still vital. Every year on March 26th, Saudi Arabia celebrates the Purple Day with activities for epilepsy patients and caregivers. Experts provide advice and seminars, media interviews, and publications to improve public awareness of and attitude towards epilepsy [43]. Health professionals, particularly community health workers, must attend annual training sessions to update their understanding of epilepsy-related concepts and best practice. To expand the number of specialists to handle more cases and lessen the workload locally, low- or middle-income countries (LMICs) could collaborate with NGOs to finance the education of more health workers. In 2006, the Mongolian Epilepsy Association organized the "Quality of Life Program" in collaboration with The First Clinical Hospital and Neurology Trainee Club. The Program includes workshops on epilepsy care for family physicians, nurses, patients, patient families, and administrators from the district health care units [44]. This educational activity was groundbreaking in enhancing the overall understanding of epilepsy. Community workers should inform their communities about PWE treatment options and how to avoid stigmatization. To keep children with epilepsy motivated, teachers should be knowledgeable about their condition and be kind to them. For PWE at work, kindness and help from employees and coworkers are important. Traditional healers should refer patients to community health workers to seek diagnosis and proper management. Conventional healers should also be educated on epilepsy [45]. Consequently, effective screening is needed for efficient identification of depression and anxiety in PWE in Asia. This can be accomplished by providing individuals with psychoeducation and psychotropic intervention. Furthermore, to reduce the prevalence of anxiety in this population, an efficient healthcare system is necessary to ensure accurate diagnosis and management of epilepsy in Asia. Improving epilepsy care and education is crucial for addressing the issue of sudden unexpected death in epilepsy (SUDEP) in Asian countries. It is vital to increase the awareness, enhance the availability of healthcare services, advance research, and foster collaborations among healthcare professionals, policymakers, and communities to prevent and reduce the occurrence of SUDEP in Asia.

SUDEP is increasingly recognized and studied in Asian countries in relation to epilepsy. SUDEP is defined as the sudden death of an individual with epilepsy that is not attributable to external factors such as trauma, drowning, status epilepticus, or other known causes. SUDEP has been associated with seizure [46], and it is a leading cause of death among individuals with epilepsy.

Due to cultural stigmas and a lack of awareness of epilepsy, many patients remain undiagnosed and untreated. The lack of access to medical care, coupled with limited knowledge about the condition, can increase the risk of SUDEP.

The incidence of SUDEP has been reported to be 1.16 cases per 1000 PWE [47]. During 2010–2019, the crude incidence rate of SUDEP was found to be 1.40% per person-year in individuals with convulsive epilepsy living in rural regions of west China [48]. Studies have identified several risk factors among Asian populations in particular, including poor seizure control, especially in individuals with medication-resistant epilepsy [49]. A study in Israel showed that the reduction of heart rate variability (HRV) is a potential biomarker for sudden cardiac death, and carbamazepine (CBZ) has been shown to have negative effects on HRV [50]. Therefore, CBZ should be used with caution in patients with known risks for SUDEP. Other factors include the duration and severity of epilepsy, age of onset, and the presence of certain comorbidities.

A combination of cultural, social, and economic factors also has a considerable impact on shaping the trends of SUDEP in Asian countries [51].

Improving epilepsy care and education is crucial to addressing the issue of SUDEP in Asian countries. Measures should be made to increase the awareness, improve the availability of healthcare services, advance research, and foster collaborations among healthcare professionals, policymakers, and communities to prevent and minimize the occurrence of SUDEP in Asia.

## Health economics of epilepsy in Asian countries

The epidemiology and treatment of epilepsy may change drastically with economic development and population aging in Asia. Nearly 80% of people with epilepsy live in low-income and middle-income nations with limited resources (Latin America, Southeast Asia, and sub-Saharan Africa) [13]. The number of new cases is twice as high as that in high-income countries. The socioeconomic risk factor may be a significant determinant of the prevalence of active epilepsy, not only in developing countries but also in rural areas of developed countries, compared to their urban counterparts [5]. A subfield of economics, "health economics", concerns the behavior of producers and consumers in the healthcare economy in a particular culture [52]. The cost per patient is high in Asian countries owing to insufficient healthcare facilities. The cost per patient in Asian regions ranges from $23 (Pakistan) to $1775 (Taiwan province of China). The per-patient costs in Malaysia, China, Singapore, and Thailand are $27, $83, $75, and $27, respectively [53]. In Asia, the direct cost on epilepsy is $625 in China over 6 months in 2009, $344 annually in India in 2001, and $361 annually in Malaysia in 2015 for patients with newly diagnosed structural-metabolic epilepsy [14].

According to an economic assessment, phenobarbital costs less than $5 per patient per year in China. People with epilepsy in low-income areas can afford healthcare visits, investigations, and travel expenses ranging from $26 to $179 [54]. Epilepsy affects the financial situation of patients' families and patients' emotional and physical states, due to the high expense of medical care and the difficulty in earning an income. The cost is affected by the diagnosis, the severity, the effectiveness of ASMs, and health insurance coverage. Due to the national health policies in Bhutan, people receive healthcare at a reduced cost, and this has encouraged families to go to hospitals for early diagnosis [55]. Japan has established a universal health insurance system with free access and low cost since 1961 [56]. A cross-sectional survey in Bhutan assessed the economic impact of epilepsy and reported indirect costs of epilepsy including negative effects on employment and school attendance. About 18% of the school-aged children participants drop out of school because of epilepsy, and this will in turn affect the economy in the future [57]. Surgery, vagus nerve stimulation (VNS), severity, and seizure control are major themes of expense. Transportation costs are a portion of the out-of-pocket epilepsy-related expenses in India and Bhutan; they impact how frequently Chinese patients go to hospitals, change ASMs, prescription refills, or continue follow-up visits. The annual direct out-of-pocket cost per patient is $372 in China and $91 in Bhutan [57].

Due to the universal health care system in Bhutan, the direct costs of epilepsy was only 3.2% of their annual income in 2018, a modest direct financial burden compared to other nations. The availability of ASMs, less stigma, and approval of Western medicine can all contribute to economic growth. In Lebanon, sociodemographic factors have a significant influence on the QoL of epileptic patients. Having a Lebanese nationality, being employed, and belonging to the medium socioeconomic level are linked to higher QoL scores [58]. This underscores the significance of financial and social stability in determining the QoL of patients. Health insurance coverage can benefit PWE by increasing hospital visits to specialized neurologists and lowering extra expenses [59]. In some nations, it has been challenging to accurately assess the economic burden of epilepsy with separation from the cost of pre-existing disorders due to the limitations in natural surveys in those countries. The only way to obtain statistics on direct or indirect costs appears to obtain self-reported patient information. Administrative data must be configured to contain comprehensive information.

## Health care services for epilepsy in Asian countries

Most communities face difficulties in managing epilepsy owing to the limited number of healthcare experts and services. In disadvantaged communities, epilepsy diagnoses are missed, morbidity and mortality increase, and PWE must travel long distances to seek help from untrained healthcare professionals [60].

Owing to the shortage of neurologists in many low-income nations, patients are managed by primary care physicians. In developed countries, there are adequate specialists, but they are mostly available in cities [61].

In low-income countries in Asia, there is 0.03 neurologist per 100,000 in average, while in high-income countries, the number is 2.96 [13]. Healthcare workers must be better trained to handle seizures. With the current crisis in Afghanistan, asylum seekers with epilepsy fear that their medical records may hinder their seeking for the refugee status. The Afghan crisis has caused more epilepsy cases in refugee camps, and fewer professionals to diagnose and treat it [62]. To meet Afghanistan's humanitarian needs, healthcare systems must expand, and healthcare workers must be trained to help during seizures. Raising awareness and reducing the stigma of epilepsy will improve the prognosis.

The availability of imaging technologies and EEG recording varies among regions, doctors in less accessible regions make diagnoses mostly based on clinical data. The mortality and morbidity rates in Asia are rising due to the failure to identify epilepsy in low-income areas [63].

There is also difficulty in interpreting EEG results in Asia because of the fewer neurologists compared to Western nations (0.07/per 100,000 in Southeast Asia to 4.84/per 100,000 in Europe) due to the high cost of establishing facilities for specialists, most of whom have completed their education abroad [13].

The use of open application programming interfaces can move data between applications as the diagnostic certainty increases. The open standard of data facilitates the exchange of information between different applications. Furthermore, it would help foster an ecosystem of patient applications that can adapt to the needs of patients over time [64]. Epilepsy could be treated in primary care facilities and epilepsy care consolidated nationwide. Improving diagnostics, identifying comorbidities, and improving referral systems are needed for epilepsy management [65]. To enhance the administration and results of epilepsy, multiple strategies can be executed, incorporating the utilization of technological breakthroughs such as remote monitoring, electronic health records, and artificial intelligence [64]. These technologies can aid in the early detection and intervention of seizures, allowing for timely and targeted treatments.

Founded in 2002 as the educational arm of the ILAE, the Asia Epilepsy Academy (ASEPA) aims to provide healthcare workers with education and training necessary for optimal epilepsy care in Asia [66], as well as providing examinations to test healthcare professionals' understanding of epilepsy, in the aim to encourage high standards of care and expertise in diagnosis, treatment, and management.

The ASEPA-ASNA (Asean Neurological Association) exam, which is developed and overseen by Asian epilepsy experts, employs a rigorous, standardized test consisting of multiple-choice questions, case-based scenarios, and practical assessments. The questions are designed to test candidates' knowledge, clinical reasoning, and decision-making abilities.

The acquisition of the ASEPA-EEG certification demonstrates a candidate's mastery in managing epilepsy in Asia. It is a widely recognized accreditation that verifies the competence and dedication of healthcare professionals in providing high-level epilepsy care. This certification closely related to career prospects and elevates the standard of epilepsy care in Asian countries.

## Treatment approaches for epilepsy in Asian countries

In low-income Asian populations, the management of epilepsy is primarily challenged by the issue of treatment. Epilepsy management can be complicated, and outcomes can be unpredictable. The primary treatment goal is to achieve seizure freedom without significant side effects [67]. With ASM treatments, seizures are controlled in 60–70% of newly treated patients. Monotherapy is widely regarded as the gold standard for initial treatment of epilepsy. If monotherapy fails, two-thirds of the experts will switch to an alternative monotherapy, whereas one-third will switch to an add-on therapy. Compared to a previous survey conducted in the United States (0%) and China (8%), a higher proportion of experts would choose add-on treatments in China [65]. Epilepsy that is not managed adequately can cause seizures, as well as an increased risk of burns and falls, potentially resulting in a mortality of 20–30% [68].

The rate of treatment in Asia has been hindered by several issues, including insufficient healthcare, lack of specialist care, limited pharmacological treatment options, lack of affordable options, distance from medical facilities, lack of awareness, stigma, beliefs, and traditional medicine. A study performed in rural regions of Vietnam found that only 15% of epilepsy patients received appropriate ASMs despite the availability of phenobarbital and phenytoin free of charge through a national program [69]. Even when using ASMs, some patients cease them without considering the risks. Phenobarbital is one of the most widely used ASMs and offers a favorable cost-benefit ratio, although the severity of its side effects remains a matter of controversy. It remains the first-line treatment in developing countries despite being abandoned in developed countries [70]. The treatment gap in epilepsy management is a major concern for patients, their families and care providers. The treatment gap is defined as the proportion of people with epilepsy who are not adequately treated [71]. The random effect means of the treatment gap are 64% in Asia (95% Cl: 24.3-100), 49% in Africa (95% Cl: 14-100), and 55.4% in Latin America (95% Cl: 39.0-78.6) [13]. The mean treatment gap prevalence in urban and rural areas is 46.8% (95% Cl: 34.1-64.8) and 73.3% (95% Cl: 49.5-100), respectively [13]. There is a higher rate of untreated epilepsy in Bangladesh (60.7%) than in China (41%). Most treatment recipients (82.5%) take antiseizure medications, and a small proportion (15.0%) use traditional medicine. The use of traditional medicine is quite high in a Chinese study (42.7%) [72].

As part of a Global Campaign Against Epilepsy project, two epidemiological surveys conducted before and after a therapy intervention in an educational program in six provinces in China showed that the treatment gap decreased from 62.6% to 49.8% (12.8% decrease, 95% Cl: 4-21.4), demonstrating that the treatment can be improved [13].

Access to ASMs can be linked to the licensing of modern epilepsy medications by a specific nation rather than a continent. ASMs may only be available in urban regions in Asia, which limits their availability [13]. Since second-generation medicines are not readily available in all nations and can be expensive, phenobarbital is still a widely used drug. Novel medications are often evaluated in a few patients to meet the regulatory standards in Europe and the USA. Population-based pharmacogenetic studies are required to ascertain the responses to novel ASM medications in Asian populations due to the genetic diversity and ethnic heterogeneity [73]. Furthermore, the COVID-19 lockdown has prevented access to ASM, while reduced healthcare services made it difficult for PWEs to maintain contact with their doctors [74].

The government should collaborate with NGOs to supply antiseizure medications and diagnostic tools to remote communities. As families continue to seek treatment from underprivileged practitioners, traditional practitioners should be included in this program [75]. Recent global COVID lockdown has made it more difficult for patients to receive follow-up care, leading to increased seizures [74]. Patients with active epilepsy should be given special care when altering or implementing other ASM dosages [76]. The Saudia Arabia government has established Epilepsy Management Units (EMUs) which are specialized treatment centers for providing quality healthcare in Saudi Arabia. Through its specialized diagnosis and treatment, the EMU offers a secure and efficient way to address the consequences of epilepsy and improve the lives of patients. This, in turn, bolsters healthcare in the nation and ultimately leads to a higher QoL for all [77]. Enhanced access to preventive and curative medications can help seizure control or management. Early identification and intervention can slow the progression of the disorder. Specialized assistance and post-diagnosis care can aid in the management of their condition.

### Epilepsy surgery: challenges of assessing appropriate candidacy and delivery effective care in Asia

For some patients with drug-resistant seizures, particularly those with temporal lobe epilepsy, surgery is a well-established treatment option [78]. A 2006 ILAE & IBE survey showed that only 13% of epilepsy surgeries were performed in low-income nations, compared to 66% in high-income nations. Significant progress has been made in several Asian countries such as India [79]. Epilepsy surgery requires expensive surgical techniques. Due to the dysfunctional primary care systems and inability to access first-line antiseizure medications, establishing neurosurgical centers is challenging in most LMICs. There is a debate over whether surgery is a cost-effective long-term investment that may benefit more people in LMICs, considering the high prevalence of epilepsy [80]. In India, there are currently 39 centers performing epilepsy surgeries, of which 18 centers were open during the past five years [79]. In India, 734 epileptics surgeries are performed annually. Due to the ongoing treatment gap, even with these advancements, only 2 out of every 1000 people are eligible for epilepsy surgery. In Qatar, there are currently many patients with drug-resistant temporal lobe epilepsy associated with hippocampal sclerosis [81]. Surgery offers an opportunity for these patients to reduce or eliminate their seizures. Neurologists with extensive knowledge of the indications, risks, and benefits of surgery are more likely to recommend it to patients than those with limited experience in the field [78].

The Association of Southeast Asian Nations (ASEAN) comprising 10 members (Brunei, Cambodia, Indonesia, Laos, Malaysia, Myanmar, the Philippines, Singapore, Thailand, and Vietnam) has 41 surgery centers, covering a 640 million population. The density of center is less than one center per 10 million people, lower than those in other regions in Asia, including Korea (14 centers 48 million people), Taiwan province in China (3 centers 23 million people), and Hong Kong of China. Most countries have pre-surgical evaluation facilities, but further invasive and noninvasive investigations are limited. Brunei, Cambodia, and East Timor are the only three ASEAN members without epilepsy centers, while only five countries have at least one level-4 epilepsy care facility Table 2 [82]. The main barriers are as follows.

1. With the lack of high-tech equipment, surgical teams have to select the most qualified candidates for surgery for the sake of safety.
2. The cost of surgery discourages patients from seeking further treatment [83].
3. The surgical treatment gap extends beyond the capabilities of epilepsy clinics [48].
4. The epilepsy program is not initiated or maintained by many trained specialists.

**Table 2:** Accessibility of epilepsy surgery in Asian countries: an analysis of the number of centers available [13]

Country	Number of epilepsy surgery centers
Brunei	0
East Timor	0
Cambodia	0
Laos	1
Nepal	1
Malaysia	4
Philippines	4
Singapore	4
Indonesia	5
Thailand	7
Myanmar	8
Vietnam	9
Korea	17
Mainland China	At least five cities in each of the 32 provinces have epilepsy centers, and at least one offers epilepsy surgery
Japan	43

The following actions should be taken under collaboration between governments and NGOs to counteract some factors:

1. The lesions of epileptogenesis should be reserved during surgery only for patients with mesial temporal lobe epilepsy and hippocampal sclerosis. In other cases, potentially epileptogenic lesions should be excised.
2. Facilities: Most countries have presurgical evaluation facilities. Surgery may be performed with consistent results of interictal EEG, neuroimaging, and seizure localization. In 45.5% of ASEAN countries, advanced noninvasive evaluations such as single-photon emission computerized tomography/positron emission tomography and invasive presurgical investigations are accessible [82]. Lack of funding prevents the establishment of epilepsy centers with innovative presurgical investigative facilities and invasive monitoring in low-income countries. Patients in Myanmar and Vietnam, where private facilities are exceedingly expensive and healthcare treatments are neither subsidized nor covered by insurance, patients are fully responsible for their costs. With sufficient financing and infrastructure, countries such as Singapore and China have more modern epilepsy centers. Certain government organizations in Asia should be encouraged to finance the patient's medical expenses.
3. An epilepsy center requires epileptologists, neurologists, neurosurgeons, and EEG specialists with extensive experience in epilepsy. Training programs for aspiring neurologists and neurosurgeons should be developed in centers [84].
4. Government agencies and NGOs should strictly implement the policies and intervention plans they have set to ease the plight of epilepsy patients within a reasonable timeframe. For example, the Belt and Road (B&R) Initiative launched by Chinese President Xi Jinping can promote communications on epilepsy research progress and experience among professionals across different countries.

### Harnessing neurostimulation for effective epilepsy control in Asian countries

The use of neurostimulation for epilepsy has been of increasing significance in Asian countries over the past few decades. Neurostimulation involves the use of electrical currents to regulate brain activity. It has become a critical treatment option for patients with drug-resistant epilepsy who failed to respond well to medications [85].

Neurostimulation therapy for epilepsy has been widely adopted and practiced. Countries in Asia have well-established healthcare systems with specialized neurology departments that provide comprehensive epilepsy care, including neurostimulation therapy [86].

One of the most common neurostimulation therapies for epilepsy is VNS. The VNS Therapy System is the first medical device therapy approved by Food and Drug Administration (FDA) for adjunctive treatment of drug-resistant epilepsy, proven with safety and tolerability [87]. In this procedure, a small generator is implanted in patient's chest, delivering electrical pulses to the vagus nerve in the neck to regulate brain activity and reduce seizure frequency [88]. An over three-year analysis of the efficacy and safety of VNS therapy in 385 drug-resistant epilepsy patients in Japan revealed a follow-up rate of over 90% at three years [89]. The study also revealed that the effectiveness of VNS therapy improved over time, up to three years. The VNS therapy has been found to be effective in reducing seizures and improving the QoL of PWE in Asian countries.

Another neurostimulation technique employed in certain Asian countries is the deep brain stimulation (DBS). DBS is a neurosurgical procedure that utilizes electrodes and a pacemaker-like device to send electrical impulses to areas of the brain [90]. Although the exact mechanism of DBS is not yet fully understood, it has been proposed that DBS provides targeted modulation of specific neural circuits within the brain. DBS was introduced in China in 1998 (source of information Beijing Tiantan Hospital). The application of DBS in China has developed rapidly since the past two decades. The number of DBS centers in China has expanded, with over 200 DBS leads implanted per year/center. However, more than 100 hospitals performed DBS in less than 10 cases per year [91]. The high costs of hardware, surgery, and follow-up care present a significant barrier to the widespread adoption of DBS.

The Asian countries have also witnessed advancements in responsive neurostimulation (RNS) therapy for epilepsy. The RNS system is a closed-loop brain stimulation system that can detect and respond to seizure activity by delivering electrical pulses [92]. It is well known that the electrical activity spreads monosynaptically and polysynaptically from a local region to other regions in the case of focal-onset epilepsy. This personalized approach allows the delivery of targeted stimulation to prevent occurrence of seizure [86]. Due to the closed-loop design, the RNS system can be used for both therapeutic treatment and understanding the molecular mechanisms of epilepsy. The closed-loop devices allow collection and archiving of long-term electrocorticogram recordings, which provide valuable data for adjustment of stimulation parameters [93].

Studies have shown positive outcomes in Asian countries after the neurostimulation therapy for epilepsy, including decreased seizure frequency, improved seizure control, and improved QoL of patients [86].

Although the application of neurostimulation for epilepsy has developed in Asian countries, problems still exist. A key barrier to various therapeutic choices is the lack of knowledge among patients, healthcare providers, and the public. Furthermore, the high expenditure of the operations and a lack of insurance coverage also hinder the wide application of neurostimulation.

### Ketogenic diet for epilepsy treatment in Asian countries

The ketogenic diet, which was first introduced in the 1920s as a treatment for epilepsy, is a high-fat, low-carbohydrate, and moderate-protein diet that aims to induce the metabolic state of ketosis in the body [94]. Ketosis is a metabolic state that occurs when the body burns fat for energy, resulting in the production of ketones as an alternative fuel source instead of glucose. This shift in metabolism is believed to reduce seizure frequency and severity in people with epilepsy [95]. Currently, there are four major types of ketogenic dietary therapy: the classic ketogenic diet, the modified Atkin diet, the medium-chain triglyceride ketogenic diet, and the low-glycemic index treatment [96]. The ketogenic diet is a therapeutic option for children with refractory epilepsy, which involves eating a high-fat, moderate-protein diet. In a recent study, about one-third of patients who stayed to the classic ketogenic diet showed more than 90% reduction in seizure frequency after 6 months [97].

Due to the variations in dietary practices and customs, the ketogenic diet should be implemented with cultural considerations in Asian countries. For instance, rice is a crucial food in many Asian countries, so its substitution by high fat can be difficult among the people. To make the diet more acceptable and sustainable in Asian communities, modified versions of the ketogenic diet have been adopted in some areas [98].

Improving the public awareness and knowledge on the advantages and applications of ketogenic diet is essential. Healthcare professionals should also have adequate training and experience on dietary administration. Services and support networks should also be provided to patients and their families for efficient nutritional intervention.

## Progress made in Asian countries

In recent years, developments on epilepsy diagnosis and treatment have been made in Asia. This progress can be attributed to increased awareness campaigns, educational programs, and the establishment of specialized epilepsy centers. Epilepsy is being accurately diagnosed among patients, enabling timely and appropriate interventions. In addition, there has been a significant development in the field of epilepsy care, with increased availability of ASMs. Several novel ASMs have been approved and introduced in Asia, so healthcare professionals have more options in hand for managing epilepsy and improving seizure control [99]. Furthermore, there has been a growing emphasis on multidisciplinary care in Asia. A multidisciplinary team consisting of neurologists, epileptologists, neurosurgeons, psychologists, and social workers will provide comprehensive care and support to individuals with epilepsy [100]. Moreover, research conducted in Asian countries has concentrated on uncovering the fundamental causes and processes of epilepsy, resulting in breakthroughs in genetics, risk factors, and potential treatments of epilepsy [101]. In addition, regulatory bodies, including the ASEPA and China Association Against Epilepsy, have been established to foster knowledge sharing, standardize practices, and encourage collaboration among healthcare professionals [102]. The progress made in the diagnosis, treatment, and multidisciplinary care of epilepsy in Asian countries demonstrates dedicated efforts to enhance the lives of those affected [103].

## Conclusions

In the last few decades, tremendous progress has been made in the treatment of epilepsy in Asia. The Research Task Force appointed by the Commission on Asian and Oceanian Affairs of the International League Against Epilepsy has outlined research priorities for the region, such as exploring the burden of epilepsy in the area, increasing access to treatment, narrowing the gap in care, gaining more insights into the causes and factors that contribute to epilepsy, including pathology, genetics, and lifestyle, and reducing the negative effects of epilepsy through disability reduction. However, the lack of data from some Asian countries, as well as the diversities in culture, socioeconomic factor, genetic background, and health infrastructure among Asian countries, present a challenge for epilepsy management. Furthermore, more collaborations between global health organizations are needed to gain a deeper understanding of the causes of epilepsy in the Asian population and provide tailored interventions.

## Data Availability

Not applicable in this section.

## Abbreviations

ASEAN: Association of Southeast Asian Nations
ASM: Antiseizure medication
ASEPA: Asian Epilepsy Academy
DBS: Deep brain stimulation
EMUs: Epilepsy management units
ILAE: International League Against Epilepsy
LMIC: Low/middle-income countries
NGO: Non-governmental organization
PWE: Patient with epilepsy
QoL: Quality of life
RNS: Responsive neurostimulation
SUDEP: Sudden unexpected death in epilepsy
UAE: United Arab Emirates
VNS: Vagus nerve stimulation
